# Validation of SpO_2_/FiO_2_ as a Non-Invasive Surrogate of PaO_2_/FiO_2_ in Mechanically Ventilated COVID-19 Patients at High Altitude

**DOI:** 10.3390/arm94030028

**Published:** 2026-04-28

**Authors:** Guillermo Ortiz-Ruiz, Manuel Garay-Fernández, Eduardo Tuta-Quintero, Alirio Bastidas, Antonio Lara, Arlen Mauricio Márquez, Carolina Aponte, Jairo Guevara, Jonathan A. Guezguan

**Affiliations:** 1 Subred Integrada de Servicios de Salud Centro Oriente ESE, Bogotá 111711, Colombia; mandres80@hotmail.com (M.G.-F.); alarag2207@yahoo.com (A.L.); mauromarquezgalindo@gmail.com (A.M.M.); jguevaraf@unal.edu.co (J.G.); alexander.guezguan@gmail.com (J.A.G.); 2 School of Medicine, Universidad de La Sabana, Campus del Puente del Común, Km. 7, Autopista Norte de Bogotá, Chía 250001, Colombia; eduardotuqu@unisabana.edu.co (E.T.-Q.); alirio.bastidas@unisabana.edu.co (A.B.); 3 Internal Medicine, Clínica Universidad de la Sabana, Chía 250001, Colombia; hermencia.aponte@clinicaunisabana.edu.co

**Keywords:** respiratory distress syndrome, COVID-19, hypoxemia, oximetry, pulse oximetry

## Abstract

**Highlights:**

**What are the main findings?**

- An SpO_2_/FiO_2_ (S/F) ratio threshold of ≥206 is an excellent predictor for identifying non-severe hypoxemia (defined as a PaO_2_/FiO_2_ ≥ 150), showing a diagnostic performance with an AUC of 0.983.
- The S/F ratio maintains its high diagnostic accuracy across various complex clinical conditions, including hypercapnia (PCO_2_ > 35 mmHg), high PEEP (>10 cmH_2_O), anemia, and hyperbilirubinemia.

**What are the implications of the main findings?**

- The S/F ratio serves as a valid, non-invasive alternative to the invasive PaO_2_/FiO_2_ ratio, allowing for the classification of respiratory distress without the need for frequent and painful arterial blood gas sampling.
- This metric is particularly beneficial for resource-limited settings and high-altitude locations where access to arterial gas analysis may be restricted, enabling faster and more cost-effective clinical decision-making.

**Abstract:**

Background: The ratio of arterial partial pressure of oxygen to fraction of inspired oxygen (PaO_2_/FiO_2_) is central to the classification of acute respiratory distress syndrome (ARDS). However, its assessment requires arterial blood gas analysis, which may be limited by availability, cost, and invasiveness. Consequently, the ratio of peripheral oxygen saturation to fraction of inspired oxygen (SpO_2_/FiO_2_) has been proposed as a non-invasive surrogate for estimating the degree of oxygenation impairment. Methods: A retrospective cross-sectional study was conducted in adult patients with COVID-19 admitted to the intensive care unit at an altitude of 2600 m above sea level (m.a.s.l.). Spearman correlation coefficients were calculated to assess the association between the SpO_2_/FiO_2_ and PaO_2_/FiO_2_ ratios and their corresponding imputation models. A generalized linear model was applied, and the diagnostic performance of the SpO_2_/FiO_2_ ratio and the imputation models for detecting severe and non-severe hypoxemia (PaO_2_/FiO_2_ cutoff value of 150) was evaluated using the area under the receiver operating characteristic curve (AUC). Results: A total of 473 patients receiving invasive mechanical ventilation were included, with a mean age of 62.4 years (SD 14.1), and a predominance of males (67.2%). An SpO_2_/FiO_2_ ratio cutoff value of ≥206 demonstrated excellent diagnostic performance, with an AUC of 0.983 (95% CI 0.97–0.99), high sensitivity (90.6%), high specificity (96.7%), and an overall correct classification rate of 93.9%. This performance remained consistent across multiple clinical scenarios. In patients with positive end-expiratory pressure > 10 cmH_2_O, the AUC was 0.982, with a specificity of 97.7%. In the presence of hyperbilirubinemia (total bilirubin ≥ 3 mg/dL), the AUC was 0.951. Among patients with hemoglobin levels < 10 g/dL, sensitivity reached 100%, although specificity was reduced. In the subgroup with arterial partial pressure of carbon dioxide > 35 mmHg, an SpO_2_/FiO_2_ ratio ≥ 206 showed near-perfect specificity (99.4%) and a positive likelihood ratio of 120.9. Conclusions: The SpO_2_/FiO_2_ ratio is a reliable and non-invasive surrogate of the PaO_2_/FiO_2_ ratio in mechanically ventilated patients with COVID-19 living at high altitude, particularly for the identification of non-severe hypoxemia.

## 1. Introduction

Acute respiratory distress syndrome (ARDS) remains a major cause of morbidity and mortality among critically ill patients worldwide and continues to pose substantial diagnostic and therapeutic challenges in contemporary critical care practice. Since its initial description by Ashbaugh and colleagues in 1967 [1], ARDS has been recognized as a highly heterogeneous clinical syndrome characterized by varying degrees of hypoxemia and lung injury. This heterogeneity underscores the need for accurate severity classification to guide therapeutic decision-making and prognostic assessment [2,3,4,5].

The Berlin definition established the arterial partial pressure of oxygen to fraction of inspired oxygen ratio (PaO_2_/FiO_2_) as the cornerstone for ARDS severity classification [6,7]. In this context, PaO_2_ represents the oxygen tension measured in arterial blood obtained through arterial blood gas (ABG) analysis, whereas FiO_2_ denotes the fraction of oxygen in the inspired gas mixture [6,7]. Despite its widespread acceptance, reliance on the PaO_2_/FiO_2_ ratio requires repeated arterial blood sampling, an invasive procedure that may be impractical in resource-limited settings or associated with patient discomfort and procedural complications [6,7].

Matthay and colleagues introduced the peripheral oxygen saturation to fraction of inspired oxygen ratio (SpO_2_/FiO_2_) as an alternative metric for ARDS classification [8]. Peripheral oxygen saturation (SpO_2_) reflects the percentage of hemoglobin saturated with oxygen, measured non-invasively by pulse oximetry, while FiO_2_ corresponds to the administered inspired oxygen fraction. The SpO_2_/FiO_2_ ratio offers a readily available and non-invasive surrogate for oxygenation assessment, potentially reducing the need for arterial blood sampling and associated healthcare costs.

Growing evidence supports the validity of the SpO_2_/FiO_2_ ratio as a surrogate for the PaO_2_/FiO_2_ ratio. Chen et al. demonstrated that ARDS classification using an SpO_2_/FiO_2_ ratio threshold of <315 yielded clinical characteristics and outcomes comparable to those obtained using a PaO_2_/FiO_2_ ratio threshold of <300 [9]. Similarly, Brown et al. reported that nonlinear imputation of PaO_2_ from SpO_2_ values resulted in lower estimation error, particularly at low PaO_2_ levels, with comparable mortality rates between patients with imputed and directly measured PaO_2_/FiO_2_ ratios [10].

Beyond its non-invasive and widely accessible nature, the SpO_2_/FiO_2_ ratio can be obtained and interpreted by healthcare personnel with minimal training [7,11]. However, its accuracy may be influenced by factors affecting pulse oximetry performance, including hyperbilirubinemia, dark skin pigmentation, and the use of high-dose vasopressors [9,12]. Although the SpO_2_/FiO_2_ ratio has been validated across diverse ARDS populations, the emergence of coronavirus disease 2019 (COVID-19) raised concerns regarding its performance in viral pneumonia-associated ARDS [13]. Subsequent studies have explored its diagnostic and prognostic utility in patients with COVID-19 [14,15]. Nevertheless, evidence in mechanically ventilated patients managed at high altitude remains scarce.

Therefore, the aim of this study was to evaluate the correlation and agreement between the SpO_2_/FiO_2_ and PaO_2_/FiO_2_ ratios in mechanically ventilated patients with COVID-19 admitted to two high-complexity centers located at 2600 m above sea level between 2020 and 2022.

## 2. Methods

### 2.1. Study Design and Setting

This was a retrospective, multicenter observational study conducted in the intensive care units (ICUs) of two tertiary-level hospitals located at an altitude of 2600 m above sea level. The study aimed to evaluate the correlation, agreement, and diagnostic performance of the SpO_2_/FiO_2_ ratio as a non-invasive surrogate of the PaO_2_/FiO_2_ ratio in patients with COVID-19 receiving invasive mechanical ventilation (IMV). This study was reported in accordance with the STROBE (Strengthening the Reporting of Observational Studies in Epidemiology) guidelines for observational research [16].

### 2.2. Participants

Adult patients (≥18 years) with laboratory-confirmed SARS-CoV-2 infection by reverse transcription polymerase chain reaction (RT-PCR), admitted to the ICU and managed with IMV, were eligible for inclusion. Patients were consecutively included during the study period. Cases with incomplete or missing paired oxygenation measurements (SpO_2_, PaO_2_, or FiO_2_) required for index calculation were excluded.

### 2.3. Variables and Data Sources

Sociodemographic, clinical, ventilatory, and laboratory data were extracted from electronic medical records. ABG parameters and ventilatory variables were collected 24 h after initiation of IMV. SpO_2_, FiO_2_, and PaO_2_ values were recorded simultaneously at the time of ABG sampling. Only temporally matched measurements were included for the calculation of oxygenation indices, ensuring direct physiological pairing between SpO_2_/FiO_2_ and PaO_2_/FiO_2_ ratios. Each patient contributed a single set of paired oxygenation measurements obtained at 24 h after initiation of IMV; no repeated measurements were included in the analysis. Severity of illness was assessed using the Sequential Organ Failure Assessment (SOFA), Acute Physiology and Chronic Health Evaluation II (APACHE II), and National Early Warning Score (NEWS). Oxygenation was evaluated using measured PaO_2_/FiO_2_ and SPO_2_/FIO_2_ ratios, as well as PaO_2_/FiO_2_ values estimated through linear, logarithmic, and nonlinear imputation models. Data were entered into a standardized Microsoft Excel^®^ database and independently verified for accuracy and completeness.

### 2.4. Statistical Analysis

Statistical analyses were performed using Stata^®^ version 17.0 (StataCorp LLC, College Station, TX, USA) [17] and Jamovi^®^ version 2.3 (The Jamovi Project, Sydney, Australia). Categorical variables were summarized as frequencies and percentages. Continuous variables were assessed for normality using the Shapiro–Wilk test and are presented as mean ± standard deviation or median with interquartile range, as appropriate.

Comparisons between patients with severe hypoxemia (PaO_2_/FiO_2_ < 150) and non-severe hypoxemia (PaO_2_/FiO_2_ ≥ 150) were performed using the chi-square or Fisher’s exact test for categorical variables, and Student’s *t*-test or Mann–Whitney U test for continuous variables, according to data distribution.

Correlation between SPO_2_/FIO_2_ and measured or imputed PaO_2_/FiO_2_ ratios was assessed using correlation coefficients and interpreted according to Mukaka’s criteria: 0–0.25 (poor), 0.26–0.50 (weak), 0.51–0.75 (moderate to strong), and 0.76–1.00 (strong to perfect) [18]. Agreement between indices was further evaluated using Bland–Altman analysis [18].

Depending on data distribution, linear regression or generalized linear models (GLM) were applied. Given the non-normal distribution of oxygenation indices, GLMs with a Gaussian family and identity link function were used to model the relationship between SpO_2_/FiO_2_ and PaO_2_/FiO_2_ ratios [19]. Receiver operating characteristic (ROC) curve analysis was performed to evaluate the diagnostic performance of SPO_2_/FIO_2_ cutoff values derived from regression models for identifying non-severe hypoxemia. Area under the curve (AUC), sensitivity, specificity, likelihood ratios, and correct classification rates were calculated. Subgroup analyses were conducted in predefined clinical scenarios, including hypercapnia (PaCO_2_ > 35 mmHg), elevated positive end-expiratory pressure (PEEP) (>10 cmH_2_O), anemia (hemoglobin < 10 g/dL), and hyperbilirubinemia (total bilirubin ≥ 3 mg/dL).

## 3. Results

A total of 473 patients under IMV were included, with a mean age of 62.4 years (SD: 14.1) and a predominance of male sex (67.2%). Ventilatory parameters showed a mean tidal volume of 441.6 mL, a PEEP of 11.4 cmH_2_O, and a plateau pressure of 23.1 cmH_2_O. More than half of the patients had severe hypoxemia (53.9%), and 78.0% required vasopressors (Table 1). Mean SOFA and NEWS scores were 5.2 and 7.5, respectively. At 24 h after IMV, the mean PaO_2_/FiO_2_ ratio was 168.5 (SD: 114.9) and the SPO_2_/FIO_2_ ratio was 224.7 (SD: 139.7), with similar values across imputation models.

When comparing patients with and without severe hypoxemia, the former were older (64.0 vs. 60.4 years; *p* = 0.004) and had higher NEWS scores (7.9 vs. 7.0; *p* = 0.002). Hematocrit was significantly lower in patients with severe hypoxemia (42.4% vs. 43.6%; *p* = 0.038). No relevant differences were observed in ventilatory parameters or most laboratory variables. At 24 h after IMV, significant differences were found in FiO_2_, pH, PaO_2_, PCO_2_, and all oxygenation indices (measured and imputed SPO_2_/FIO_2_ and PaO_2_/FiO_2_), with consistently lower values in the severe hypoxemia group (*p* < 0.01) (Table 2).

An SPO_2_/FIO_2_ cutoff ≥206 achieved an AUC of 0.983 (95% CI: 0.97–0.99), with high sensitivity (90.6%), specificity (96.7%), and correct classification of 93.9% (Table 3). This performance remained consistent across different clinical scenarios (Table 3). In patients with PEEP > 10 cmH_2_O, the AUC was 0.982 with a specificity of 97.7%. In the presence of hyperbilirubinemia (≥3 mg/dL), the AUC was 0.951, while in patients with hemoglobin < 10 g/dL, sensitivity reached 100%, albeit with lower specificity. In the subgroup with PCO_2_ > 35 mmHg, an SPO_2_/FIO_2_ ≥206 showed near-perfect specificity (99.4%) and a high positive likelihood ratio (LR+) of 120.9.

**Figure 1:**
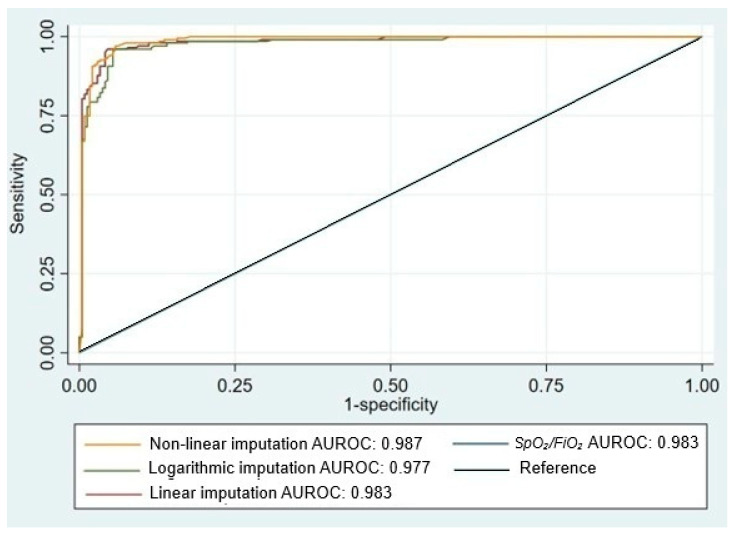
Performance of SpO_2_/FiO_2_ and PaO_2_/FiO_2_ models obtained through linear, logarithmic, and nonlinear imputation for the diagnosis of non-severe hypoxemia. Notes: Receiver operating characteristic (ROC) curve showing the diagnostic performance of the SpO_2_/FiO_2_ ratio for identifying non-severe hypoxemia (PaO_2_/FiO_2_ ≥ 150). The area under the curve (AUC) is displayed with corresponding 95% confidence intervals. The diagonal reference line represents no discriminatory ability.

**Figure 2:**
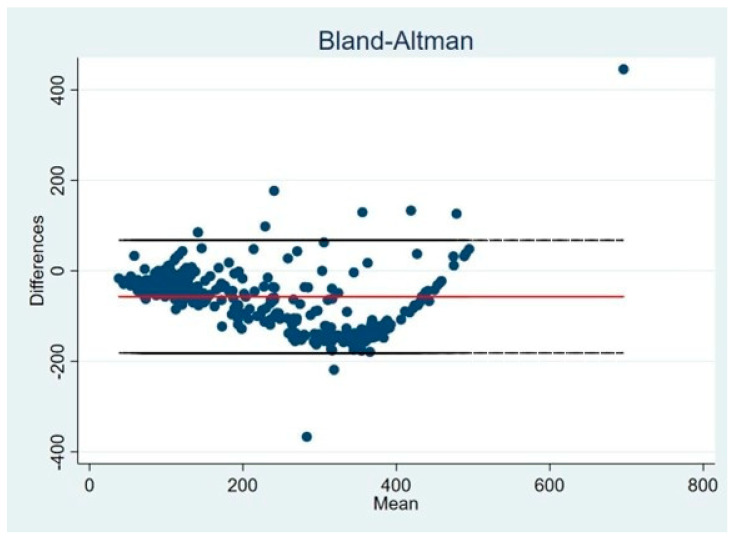
Agreement between SpO_2_/FiO_2_ and PaO_2_/FiO_2_ ratios assessed by Bland–Altman analysis. Notes: Bland–Altman plot illustrating the agreement between SpO_2_/FiO_2_ and PaO_2_/FiO_2_ ratios. The solid horizontal line represents the mean difference (bias), while the dashed lines indicate the 95% limits of agreement (mean difference ± 1.96 standard deviations). The zero reference line is included to facilitate interpretation of systematic bias.

Because oxygenation indices showed non-normal distributions, generalized linear models were applied. Distinct equations were derived for PaO_2_/FiO_2_ ≥150 and <150, identifying an SPO_2_/FIO_2_ threshold of approximately 206 to correspond with a PaO_2_/FiO_2_ of 150. Correlations between SPO_2_/FIO_2_ and measured or imputed PaO_2_/FiO_2_ were high across severe and non-severe hypoxemia. The SPO_2_/FIO_2_ ratio demonstrated excellent discrimination of non-severe hypoxemia (AUC 0.983; LR^+^ 27.4; LR^−^ 0.09) (Figure 1). Bland–Altman analysis showed minimal mean bias and acceptable agreement, particularly in intermediate oxygenation ranges, with increasing dispersion at extreme values, indicating heteroscedasticity and reduced precision in very severe or very high oxygenation states (Figure 2).

## 4. Discussion

This study evaluated the diagnostic performance of the SPO_2_/FIO_2_ ratio as a non-invasive surrogate of the PaO_2_/FiO_2_ ratio in mechanically ventilated patients with COVID-19-related ARDS treated at an altitude of 2600 m above sea level. Our findings demonstrate a strong correlation between SPO_2_/FIO_2_ and PaO_2_/FiO_2_ ratios, both when PaO_2_/FiO_2_ was directly measured using arterial blood gases and when estimated through linear, logarithmic, and nonlinear imputation models. Importantly, the SPO_2_/FIO_2_ ratio showed excellent performance in identifying non-severe hypoxemia, maintaining high discriminative ability across relevant clinical scenarios such as hypercapnia, elevated PEEP, anemia, and hyperbilirubinemia, with AUC values consistently above 0.90 in most subgroups.

These results are consistent with previously published studies conducted in different geographic and clinical contexts. Bonaventura et al. reported a strong correlation between SPO_2_/FIO_2_ and PaO_2_/FiO_2_ ratios in a cohort of 1028 hospitalized patients with COVID-19 in Italy, with excellent accuracy for identifying both mild and severe ARDS (AUC 0.958 and 0.973, respectively) [20]. Despite differences in population characteristics and care settings—our cohort exclusively included patients under invasive mechanical ventilation, whereas Bonaventura et al. studied hospitalized patients—the concordance of findings supports the robustness of the SPO_2_/FIO_2_ ratio as a reliable surrogate for PaO_2_/FiO_2_ in COVID-19-related ARDS.

The clinical utility of the SPO_2_/FIO_2_ ratio has been questioned in prior studies due to concerns regarding agreement with the PaO_2_/FiO_2_ ratio and a tendency to overestimate hypoxemia severity, potentially influencing clinical decision-making. However, such observations largely derive from retrospective database studies that included heterogeneous ARDS populations unrelated to COVID-19 and conducted predominantly at sea level [15,20,21]. In contrast, our study specifically focused on a homogeneous cohort of patients with COVID-19-related ARDS managed at high altitude, where physiological differences in oxygenation are expected. Bland–Altman analysis in our cohort showed minimal mean bias and acceptable agreement, particularly within intermediate oxygenation ranges, supporting the clinical applicability of the SPO_2_/FIO_2_ ratio in this context.

A key finding of our analysis was the consistent predictive performance of the SPO_2_/FIO_2_ ratio across diverse clinical conditions known to affect oxygenation indices. This aligns with prior evidence from Zhang et al., who demonstrated an association between the SPO_2_/FIO_2_ ratio and the need for invasive mechanical ventilation in non-ICU settings [11]. Additionally, Ruangsomboon et al. reported that the SPO_2_/FIO_2_ ratio outperformed the ROX index in predicting high-flow nasal cannula failure in patients with COVID-19 [21]. This superiority may be explained by the phenomenon of “silent hypoxemia,” frequently observed in COVID-19, where respiratory rate may remain relatively preserved despite severe hypoxemia. The SPO_2_/FIO_2_ ratio, by focusing solely on oxygenation, avoids this limitation.

The validation of the SpO_2_/FiO_2_ ratio at 2600 m above sea level is particularly relevant, as reduced barometric pressure leads to lower inspired oxygen tension and physiological adaptations such as chronic hypoxemia and shifts in the oxyhemoglobin dissociation curve [22,23,24,25]. These factors may alter the relationship between SpO_2_ and PaO_2_ compared with sea-level conditions, potentially affecting the performance and calibration of SpO_2_/FiO_2_ thresholds, as a given SpO_2_ value may correspond to a lower PaO_2_ due to changes in hemoglobin saturation dynamics and compensatory mechanisms [22,23,24,25]. While our findings demonstrate robust diagnostic performance in a high-altitude population, caution is warranted when extrapolating these thresholds to sea-level environments, where oxygenation physiology differs [24,25]. Moreover, this study reflects the clinical reality of many middle- and low-income countries, where access to arterial blood gas analysis and ICU resources may be limited.

In the contemporary pathophysiology of ARDS, oxygenation indices should not be interpreted in isolation from ventilator-induced lung injury (VILI) mechanisms and patient–ventilator interactions [26,27]. As highlighted by Merola and colleagues, barotrauma, volutrauma, atelectrauma, and biotrauma arise from heterogeneous lung mechanics and may decouple simple oxygenation measures from the true burden of lung stress and strain, particularly in patients with severe ARDS [26,27]. Therefore, exclusive reliance on global or surrogate indices, without accounting for individual respiratory mechanics, may overlook key determinants of oxygenation and lung injury, reinforcing the need for an individualized interpretation of S/F and P/F relationships [26,27,28]. Our findings should be interpreted with caution when extrapolating these cut-off values to sea-level settings, where both oxygenation physiology and ventilator–lung interactions may differ.

### Limitations

The retrospective design introduces the potential for selection bias and limits control over unmeasured confounders. No formal sample size or power calculation was performed; however, the relatively large sample size likely provided adequate statistical power for the primary analyses, as suggested by the precision of the estimates and narrow confidence intervals. Data on ventilatory management strategies, PEEP titration, the use of the prone position and interventions targeting anemia or hyperbilirubinemia were not available, which may have influenced oxygenation indices. Although statistical adjustments were applied, residual confounding cannot be excluded.

The assessment of SPO_2_/FIO_2_ performance across clinically relevant subgroups allows for a nuanced interpretation of its diagnostic utility. The use of generalized linear models allowed for robust analysis in the presence of non-normal distributions and heteroscedasticity while preserving the interpretability of coefficients on the original scale. Additionally, the use of ROC curve analysis with confidence intervals provided a robust quantitative assessment of diagnostic performance, particularly for identifying non-severe hypoxemia. Additionally, the comparison across multiple PaO_2_/FiO_2_ imputation methods adds methodological rigor, demonstrating consistent correlations regardless of the estimation approach.

No internal validation methods or external validation cohort were applied, which may increase the risk of overfitting and limit the generalizability of the proposed SpO_2_/FiO_2_ threshold. Finally, the homogeneity of the population in terms of altitude and ARDS etiology restricts generalizability to non-COVID-19 ARDS or populations at sea level. Therefore, prospective multicenter studies across different altitudes and etiologies are warranted to confirm these findings and evaluate their impact on clinically meaningful outcomes.

## 5. Conclusions

The SpO_2_/FiO_2_ ratio appears to be a reliable, non-invasive surrogate of the PaO_2_/FiO_2_ ratio in mechanically ventilated COVID-19 patients at 2600 m above sea level, particularly for identifying non-severe hypoxemia. Its consistent performance across multiple clinical scenarios suggests potential utility in resource-limited settings. However, these findings should be interpreted with caution, as the proposed cutoff requires validation in independent cohorts. PaO_2_/FiO_2_ remains the reference standard in severe hypoxemia, and arterial blood gases should be prioritized in these cases.

## Figures and Tables

**Table 1:** Sociodemographic, clinical, and ventilatory characteristics.

Total, *n* (%)	473 (100)
Age, years, mean (SD)	62.37 (14.05)
Male sex, *n* (%)	318 (67.23)
Systolic blood pressure, mmHg, mean (SD)	123.49 (23.67)
Diastolic blood pressure, mmHg, mean (SD)	72.15 (14.49)
Heart rate, beats/min, mean (SD)	96.14 (18.97)
Respiratory rate, breaths/min, mean (SD)	23.19 (7.29)
Temperature, °C, mean (SD)	36.57 (1.23)
Tidal volume, mL, mean (SD)	441.64 (64.20)
PEEP, cmH_2_O, mean (SD)	11.40 (2.45)
Plateau pressure, cmH_2_O, mean (SD)	23.05 (5.49)
Peak pressure, cmH_2_O, mean (SD)	28.64 (6.32)
Driving pressure, cmH_2_O, mean (SD)	10.79 (5.09)
Dynamic compliance, mL/cmH_2_O, mean (SD)	29.19 (15.13)
Static compliance, mL/cmH_2_O, mean (SD)	43.59 (31.35)
Inspiratory airway resistance, cmH_2_O/L/s, mean (SD)	13.99 (6.16)
Leukocytes, ×10^9^/L, mean (SD)	11,418.95 (12,036.43)
Hemoglobin, g/dL, mean (SD)	14.34 (2.32)
Hematocrit, %, mean (SD)	42.94 (6.56)
Platelets, ×10^9^/L, mean (SD)	238,066.31 (96,186.23)
Total bilirubin, mg/dL, mean (SD)	0.88 (1.17)
Creatinine, mg/dL, mean (SD)	1.49 (2.08)
Sodium, mmol/L, mean (SD)	137.03 (5.40)
Potassium, mmol/L, mean (SD)	4.42 (0.87)
D-dimer, ng/mL, mean (SD)	2276.07 (4907.27)
Troponin, ng/mL, mean (SD)	66.46 (262.33)
Lactate dehydrogenase, U/L, mean (SD)	589.48 (663.18)
SOFA score, m (SD)	5.22 (2.73)
NEWS score, m (SD)	7.46 (3.15)
Use of vasopressors, *n* (%)	369 (78.01)
Arterial blood gas parameters of patients 24 h after IMV in the ICU	
FiO_2_, fraction, mean (SD)	57.34 (32.21)
pH, mean (SD)	7.38 (0.11)
PaCO_2_, mmHg, mean (SD)	37.71 (13.00)
PaO_2_, mmHg, mean (SD)	67.19 (23.48)
HCO_3_^−^, mmol/L, mean (SD)	21.71 (4.93)
SaO_2_, %, mean (SD)	88.53 (10.47)
SpO_2_/FiO_2_ ratio, mean (SD)	224.66 (139.65)
PaO_2_/FiO_2_ ratio, mean (SD)	168.45 (114.91)

Notes: SD, standard deviation; PEEP, positive end-expiratory pressure; SOFA, Sequential Organ Failure Assessment; NEWS, National Early Warning Score; IMV, invasive mechanical ventilation; ICU, intensive care unit; FiO_2_, fraction of inspired oxygen; PaCO_2_, arterial carbon dioxide pressure; PaO_2_, arterial oxygen pressure; HCO_3_^−^, bicarbonate; SaO_2_, arterial oxygen saturation; SpO_2_, peripheral oxygen saturation.

**Table 2:** Sociodemographic, clinical, ventilatory, and laboratory characteristics adjusted for mortality in patients under mechanical ventilation.

	Severe Hypoxemia *n* = 255 (53.91)	No severe Hypoxemia *n* = 218 (46.09)	*p* Value
Age, years, mean (SD)	64.04 (13.68)	60.40 (14.24)	0.004 *
Male sex, *n* (%)	168 (65.88)	150 (68.81)	0.499
Systolic blood pressure, mmHg, mean (SD)	122.35 (22.76)	124.82 (24.68)	0.272
Diastolic blood pressure, mmHg, mean (SD)	71.35 (14.00)	73.08 (15.02)	0.210
Heart rate, beats/min, mean (SD)	94.69 (17.94)	97.76 (19.97)	0.085
Respiratory rate, breaths/min, mean (SD)	23.74 (7.93)	22.55 (6.46)	0.085
Temperature, °C, mean (SD)	36.56 (1.49)	36.59 (0.84)	0.801
Tidal volume, mL, mean (SD)	439.67 (57.99)	446.60 (77.92)	0.430
PEEP, cmH_2_O, mean (SD)	11.39 (2.48)	11.44 (2.39)	0.848
Plateau pressure, cmH_2_O, mean (SD)	23.45 (5.29)	21.86 (6.05)	0.243
Peak pressure, cmH_2_O, mean (SD)	28.73 (6.59)	28.36 (5.58)	0.814
Driving pressure, cmH_2_O, mean (SD)	11.09 (4.93)	9.86 (5.69)	0.435
Dynamic compliance, mL/cmH_2_O, mean (SD)	28.59 (15.85)	31.53 (12.16)	0.505
Static compliance, mL/cmH_2_O, mean (SD)	44.47 (35.41)	40.89 (12.98)	0.676
Inspiratory airway resistance, cmH_2_O/L/s, mean (SD)	14.97 (6.40)	11.40 (4.79)	0.101
Leukocytes, ×10^9^/L, mean (SD)	11,052.27 (5077.75)	11,852.14 (16,911.55)	0.474
Hemoglobin, g/dL, mean (SD)	14.19 (2.09)	14.51 (2.57)	0.133
Hematocrit, %, mean (SD)	42.36 (5.86)	43.62 (7.27)	0.038 *
Platelets, ×10^9^/L, mean (SD)	244,784.60 (96,123.68)	230,017.00 (9567.77)	0.099
Total bilirubin, mg/dL, mean (SD)	0.80 (0.71)	0.99 (1.54)	0.119
Creatinine, mg/dL, mean (SD)	1.52 (2.41)	1.44 (1.60)	0.692
Sodium, mmol/L, mean (SD)	136.78 (5.12)	137.36 (5.74)	0.274
Potassium, mmol/L, mean (SD)	4.39 (0.90)	4.51 (0.76)	0.618
D-dimer, ng/mL, mean (SD)	1980.78 (4883.27)	2605.56 (4925.87)	0.203
Troponin, ng/mL, mean (SD)	53.58 (116.73)	80.94 (361.83)	0.303
Lactate dehydrogenase, U/L, mean (SD)	640.73 (857.21)	533.73 (339.25)	0.105
SOFA score, m (SD)	5.43 (2.76)	4.98 (2.68)	0.122
NEWS score, m (SD)	7.93 (3.09)	6.95 (3.14)	0.002 *
Use of vasopressors, *n* (%)	197 (77.25)	172 (78.90)	0.667
Arterial blood gas parameters of patients 24 h after IMV in the ICU			
FiO_2_, fraction, mean (SD)	82.50 (20.42)	27.90 (12.43)	0.001 *
pH, mean (SD)	7.36 (0.11)	7.39 (0.10)	0.002 *
PaCO_2_, mmHg, mean (SD)	40.75 (13.01)	34.18 (12.10)	0.001 *
PaO_2_, mmHg, mean (SD)	64.12 (19.27)	70.77 (27.21)	0.002 *
HCO_3_^−^, mmol/L, mean (SD)	22.45 (4.38)	20.85 (5.38)	0.001 *
SaO_2_, %, mean (SD)	87.76 (11.14)	89.44 (9.57)	0.090
SpO_2_/FiO_2_ ratio, mean (SD)	115.01 (43.52)	355.37 (95.03)	0.001 *
PaO_2_/FiO_2_ ratio, mean (SD)	82.15 (27.61)	269.40 (94.02)	0.001 *

Notes: SD, standard deviation; PEEP, positive end-expiratory pressure; SOFA, Sequential Organ Failure Assessment; NEWS, National Early Warning Score; IMV, invasive mechanical ventilation; ICU, intensive care unit; FiO_2_, fraction of inspired oxygen; PaCO_2_, arterial carbon dioxide pressure; PaO_2_, arterial oxygen pressure; HCO_3_^−^, bicarbonate; SaO_2_, arterial oxygen saturation; SpO_2_, peripheral oxygen saturation; *, *p*-value less than 0.05.

**Table 3:** Performance of the SF ratio for the diagnosis of non-severe hypoxemia in patients under mechanical ventilation across different clinical scenarios.

Variable	AUC (IC 95%)	Sensitivity (%)	Specificity (%)	LR (+)	LR (−)	Correctly Classified (%)
SpO_2_/F_i_O_2_ ≥ 206 ^a^	0.983 (0.97–0.99)	90.64	96.69	27.42	0.09	93.93
SpO_2_/F_i_O_2_ ≥ 206 (PEEP > 10)	0.982 (0.97–0.99)	78.79	97.70	34.28	0.22	92.50
SpO_2_/F_i_O_2_ ≥ 206 ^b^	0.951 (0.90–0.99)	91.67	92.73	12.60	0.09	92.23
SpO_2_/F_i_O_2_ ≥ 206 Hemoglobin < 10 g/dL	0.906 (0.72–1.00)	100.00	75.00	4.00	0.01	90.00
SpO_2_/F_i_O_2_ ≥ 206 PaCO_2_ > 35 mmHg	0.976 (0.95–0.99)	74.67	99.38	120.96	0.25	91.56

Notes: AUC, area under the receiver operating characteristic curve; 95% CI, 95% confidence interval; SpO_2_, peripheral oxygen saturation measured by pulse oximetry; FiO_2_, fraction of inspired oxygen; SpO_2_/FiO_2_, peripheral oxygen saturation to fraction of inspired oxygen ratio; PEEP, positive end-expiratory pressure; PaCO_2_, arterial partial pressure of carbon dioxide; LR(+), positive likelihood ratio; LR(−), negative likelihood ratio. ^a^: Non-severe hypoxemia; ^b^: Total bilirubin ≥3 mg/dL.

## Data Availability

The original contributions presented in this study are included in the article. Further inquiries can be directed to the corresponding author.
